# The pioneer factor PBX1 is a novel driver of metastatic progression in ERα-positive breast cancer

**DOI:** 10.18632/oncotarget.4243

**Published:** 2015-06-15

**Authors:** Luca Magnani, Darren K. Patten, Van T.M. Nguyen, Sung-Pil Hong, Jennifer H. Steel, Naina Patel, Ylenia Lombardo, Monica Faronato, Ana R. Gomes, Laura Woodley, Karen Page, David Guttery, Lindsay Primrose, Daniel Fernandez Garcia, Jacqui Shaw, Patrizia Viola, Andrew Green, Christopher Nolan, Ian O. Ellis, Emad A. Rakha, Sami Shousha, Eric W.-F. Lam, Balázs Győrffy, Mathieu Lupien, R. Charles Coombes

**Affiliations:** ^1^ Department of Surgery and Cancer, Imperial College London, London, UK; ^2^ Department of Cancer Studies, University of Leicester, Leicester, UK; ^3^ Laboratory of Medicine, Histopathology Department, Royal Brompton Hospital, London, UK; ^4^ Division of Cancer and Stem Cells, School of Medicine, University of Nottingham, Budapest, HU; ^5^ MTA TTK Lendület Cancer Biomarker Research Group, 2nd Department of Pediatrics, Semmelweis University and MTA-SE Pediatrics and Nephrology Research Group, Budapest, HU; ^6^ The Princess Margaret Cancer Centre, University Health Network, Toronto, ON, Canada; ^7^ Department of Medical Biophysics, University of Toronto, Toronto, ON, Canada

**Keywords:** breast cancer, estrogen receptor, drug resistance, metastasis, pioneer factors

## Abstract

Over 30% of ERα breast cancer patients develop relapses and progress to metastatic disease despite treatment with endocrine therapies. The pioneer factor PBX1 translates epigenetic cues and mediates estrogen induced ERα binding. Here we demonstrate that PBX1 plays a central role in regulating the ERα transcriptional response to epidermal growth factor (EGF) signaling. PBX1 regulates a subset of EGF-ERα genes highly expressed in aggressive breast tumours. Retrospective stratification of luminal patients using PBX1 protein levels in primary cancer further demonstrates that elevated PBX1 protein levels correlate with earlier metastatic progression. In agreement, PBX1 protein levels are significantly upregulated during metastatic progression in ERα-positive breast cancer patients. Finally we reveal that PBX1 upregulation in aggressive tumours is partly mediated by genomic amplification of the PBX1 locus. Correspondingly, ERα-positive breast cancer patients carrying PBX1 amplification are characterized by poor survival. Notably, we demonstrate that PBX1 amplification can be identified in tumor derived-circulating free DNA of ERα-positive metastatic patients. Metastatic patients with PBX1 amplification are also characterized by shorter relapse-free survival. Our data identifies PBX1 amplification as a functional hallmark of aggressive ERα-positive breast cancers. Mechanistically, PBX1 amplification impinges on several critical pathways associated with aggressive ERα-positive breast cancer.

## INTRODUCTION

Over 30% of ERα-positive breast cancer patients treated with endocrine therapies eventually relapse and progress into metastatic disease [1]. Previous studies strongly suggest that the endocrine therapy resistance and metastatic progression occur in a multi-step manner and are driven by genetic and epigenetic alterations [2–4]. Several evidences indicate that breast cancer growth becomes ultimately independent from ERα [5–8]. Nonetheless this may be occurring toward the final stages of tumour progression. For instance, recent studies have discovered the existence of ERα activating mutations specifically in metastatic patients [9–12]. Additionally, ERα can be activated in the absence of estrogen by alternative mitogens including the epidermal growth factor (EGF) [13, 14]. EGF promotes ERα binding to thousands of regulatory elements and activates the transcription of a set of genes commonly over-expressed in the HER2 subtype and associated with disease progression [15].

We have recently described the role of PBX1 as a novel pioneer factor in ERα breast cancer [16]. PBX1 is critical to maintain chromatin accessibility and allow estrogen induced ERα recruitment to thousands of regulatory elements [16]. More importantly, we demonstrated that PBX1 is necessary for the transcriptional regulation of a subset of estrogen target genes directly associated with poor outcome. Furthermore, we have demonstrated that PBX1 upregulation is involved in the development of endocrine therapy resistance *in vitro* and that mRNA levels of genes controlled via Notch-PBX1 crosstalk are associated with poor prognosis in ERα patients [5]. Whether PBX1 contributes to estrogen-independent, EGF driven activation and recruitment of ERα is not yet known. We have thus investigated the role of PBX1 in mediating EGF signalling in ERα-positive breast cancer cell lines using genome wide-analyses including microarray and ChIP-seq. Our results demonstrate that PBX1 is required to direct EGF-ERα signalling at the chromatin level in breast cancer cells. PBX1 is also required for the expression of genes associated with tumour progression. PBX1 protein levels were significantly upregulated *in vivo* in metastatic samples from patients treated with endocrine therapies. Analysis of an independent historical collection of primary samples demonstrated that PBX1 protein levels correlate with shorter time to progression in ERα patients. Finally, we present evidences suggesting that PBX1 may be amplified in over 10% of ERα-positive breast cancer patients and amplification is associated with shorter survival. PBX1 amplification was confirmed in the circulating free DNA (cfDNA) of metastatic patients further suggesting the potential for using PBX1 to monitor breast cancer progression.

## RESULTS

### PBX1 mediates EGF signaling in ERα-positive breast cancer cells

**Figure 1:**
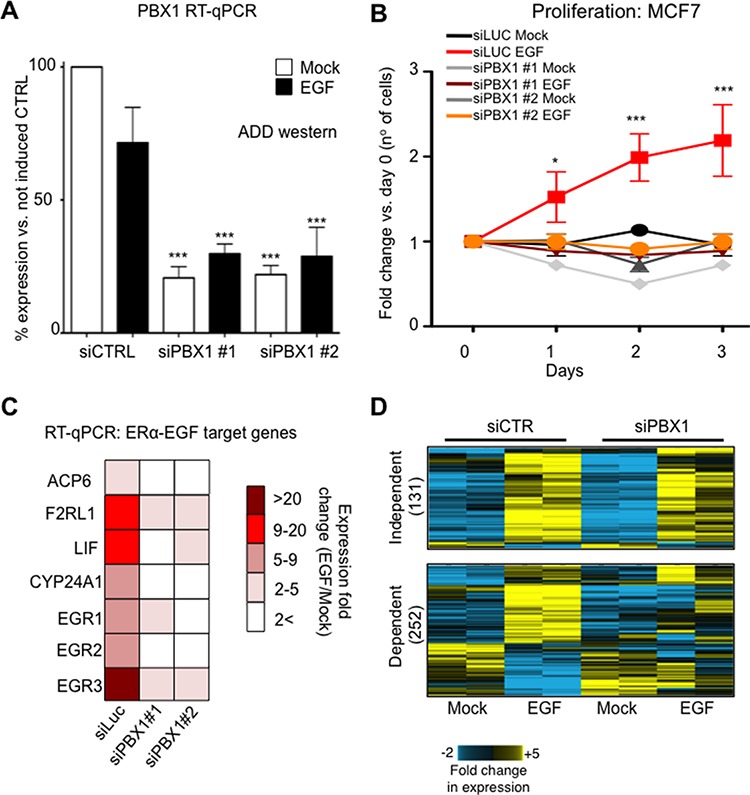
PBX1 is required to activate EGF signaling in ERα breast cancer cells **A.** PBX1 silencing using two independent siRNAs **B.** Proliferation curves in response to EGF stimulation (100 ng/ml) or mock control in MCF7 cells. **C.** Transcriptional activation of several EGF target genes is lost upon PBX1 depletion. The fold induction was calculated averaging three independent experiments. All comparison between siRNA and control are statically significant (Student's *t*-test, *p* < 0.05) **D.** Microarray analysis of PBX1 depleted cells demonstrates that PBX1 control a large portion of EGF dependent genes. Asterisks identify significant differences between treatments.

We recently demonstrated that PBX1 is a pioneer factor in ERα-positive breast cancer, occupying the chromatin prior to ERα binding following its activation with estrogen and regulating the expression of estrogen dependent genes associated with aggressive progression [16]. In addition we showed that ERα controls a different transcriptional program when activated by growth factors [15]. Specifically, EGF can activate ERα via serine phosporylation and promotes transcription of genes associated with aggressive HER2 positive tumours [15]. Therefore, we reasoned that PBX1 might also play a role in estrogen independent EGF driven ERα induced transcription. ERα-positive MCF7 cells proliferate in response to EGF when cultured in the absence of estrogen and this phenotype is dependent on ERα [15]. PBX1 depletion using two siRNA molecules is sufficient abrogate the proliferative response induced by EGF suggesting that PBX1 regulates EGF-ERα signaling (Fig. 1A–1B). Similar results were also obtained using T47-D, a second independent ERα-positive breast cancer cell line (Fig. S1A). We and others have previously showed that PBX1 is a direct Notch target that can be potentially antagonized with gamma secretase inhibitors (GSI, MRK003 and PF03084014) [5, 17]. In agreement, treatment with MRK003 is sufficient to downregulate PBX1 at mRNA and protein levels (Fig. S1B–S1C) and suppresses EGF induced proliferation in MCF7 cells (Fig. S1D–S1E). EGF activates ERα by promoting receptor phosporylation at several key residues via AKT signaling pathway [15], including S118 [18]. Notably, PBX1 silencing did not impair ERα phosphorylation suggesting that defects in EGF induced proliferation did not stem from defect in non-canonical activation of ERα [19] (Fig. S2A). However, growth deficit in response to GSI treatment might stem from a combination of PBX1 downregulation and direct inhibition of EGF signalling (Fig. S2A).

**Figure 2:**
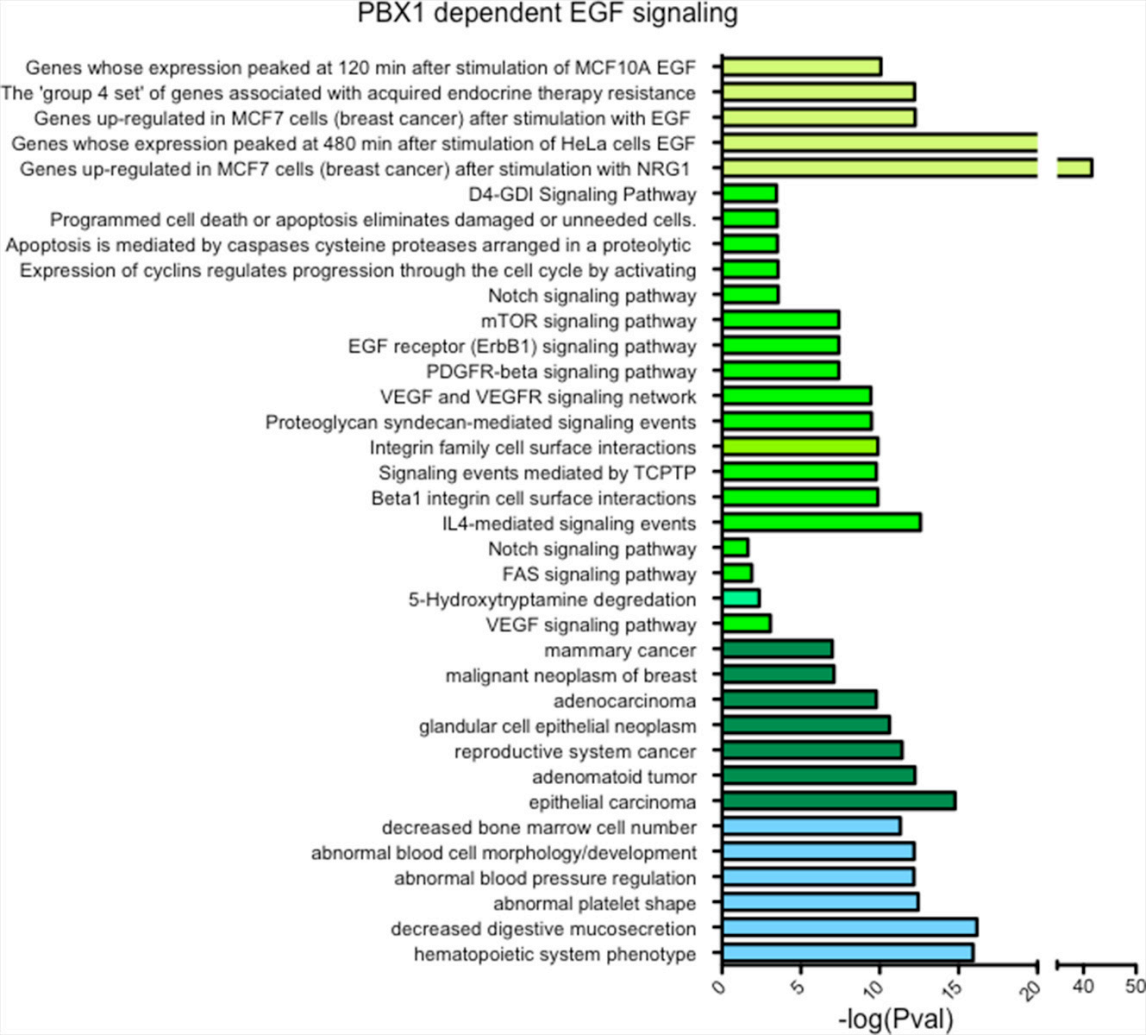
PBX1 controls the expression of genes associate with breast cancer progression Genes whose failed to be upregulated by EGF upon PBX1 silencing (*n* = 147) were used for ontology analysis using GREAT [48].

Next, we investigated the transcriptional effect of PBX1 silencing on the EGF-ERα transcriptional program using qRT-PCR. As expected, silencing PBX1 in MCF7 cells impaired the transcriptional activation of a known set of EGF-ERα target genes [15] (Fig. 1C). These results were confirmed and expanded using expression microarrays (Fig. 1D). Our data indicate that over 60% (252 out of 383) of the genes differentially expressed in response to EGF signaling require PBX1 for their regulation (Fig. 1D and Table S1). These data are strongly reminiscent of our previous finding in which a subset of estrogen responsive genes were dependent on PBX1 [16]. PBX1 dependent genes were enriched for several important ontological terms associated with breast cancer and endocrine therapy resistance, including Notch signaling, pro-invasive signaling and epithelial and mammary carcinoma (Fig. 2). Interestingly, genes that failed to be induced by EGF in PBX1 depleted cells are also significantly enriched in several independent genes datasets obtained from patients characterized by aggressive breast cancers (Fig. S2B). Overall, these data suggest that PBX1 underlies the expression of EGF dependent genes involved in aggressive tumour progression.

### PBX1 is pre-loaded at regulatory elements recruiting ERα during EGF signaling

**Figure 3:**
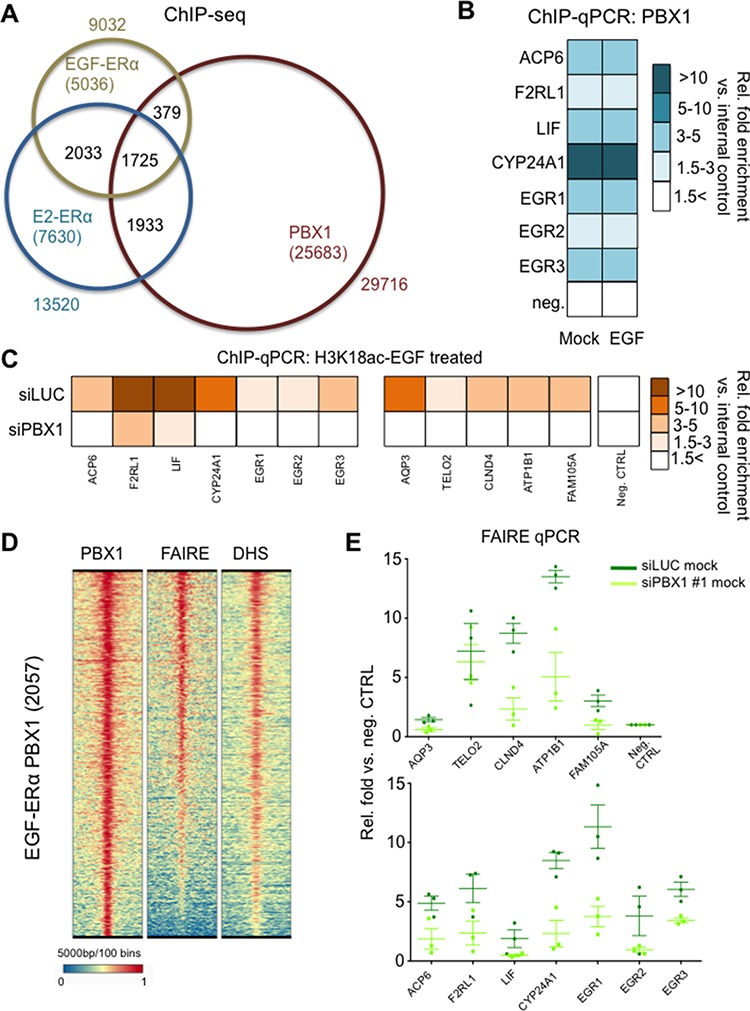
PBX1 regulate chromatin status at EGF-ERα binding sites **A.** ChIP-seq analysis of PBX1 and ERα in MCF7 cells **B.** ChIP-qPCR validation of PBX1 binding at potential regulatory EGF-ERα binding sites in the absence of EGF stimulation. CTRL indicate stimulation using mock. The fold enrichment was calculated averaging three independent experiments. All comparison between siRNA and control are not statically significant (Student's *t*-test, *p* < 0.050) **C.** PBX1 silencing abrogates epigenetic co-activation of EGF-ERα regulatory elements as measured by induced H3K18 acetylation. The fold enrichment was calculated averaging three independent experiments. All comparison between siRNA and control are statically significant (Student's *t*-test, *p* < 0.050) **D.** Genome wide chromatin accessibility analyses at PBX1 and EGF-ERα shared sites. **E.** PBX1 depletion negatively impact chromatin accessibility at EGF-ERα binding sites as identified by reduced FAIRE signal.

Pioneer factors such as PBX1 and FOXA1 bind to the chromatin prior to ERα recruitment where they are thought to mediate or maintain chromatin accessibility [20–22]. To determine if PBX1 could serve as a pioneer factor for ERα under EGF stimulation we conducted ChIP-seq assays in MCF7 cells. We then compared the EGF-ERα cistrome (e.g. ERα genome wide binding sites induced upon EGF stimulation) with the estrogen-ERa cistrome and previously published PBX1 cistrome. In agreement with previous reports [15], most ERα chromatin binding is unique to specific stimuli (EGF-ERα vs. E2-ERα) (Fig. 3A and S3A). PBX1 occupies approximately 30% of ERα binding sites, regardless of the stimulus (Fig. 3A). PBX1 binding occurs prior and is not affected by EGF stimulation as demonstrated by direct ChIP-qPCR assays at selected binding sites in proximity of EGF regulated genes (Fig. 3B). This process is strongly reminiscent of PBX1 behaviour in the context of estrogen stimulation [16]. We next considered if EGF induced ERα binding sites that overlap with PBX1 have a functional relationship with the EGF-ERα transcriptional program. To do this we have combined transcriptional and ChIP-seq data of MCF-7 cells treated with EGF. We have analysed genes whose expression changed in response to EGF stimulation (all upregulated genes) for enriched binding patterns near their transcriptional starting site (up to 20 kb upstream of the TSS). We focused on ERα binding in response to estrogen (17 beta-estradiol, E2) or EGF with or without concurrent PBX1 and FOXA1 binding. Our data show that genes that respond to EGF are significantly enriched for PBX1-EGF-ERα binding but lack PBX1-E2-ERα binding sites (Fig. S3B, red dots). FOXA1 binding was less enriched near these genes (Fig. S3B, light-blue dots). EGF responsive genes are also significantly enriched for PBX1-FOXA1-non specific ERα binding, suggesting that co-activation may also play an important role to regulate this subset of genes (Fig. S3, purple dots). As expected, regions pre-bound by pioneer factors that did not recruit ERα lacked any association with transcription. Collectively, these analyses confirm that PBX1 binding is directly involved in regulating EGF-ERα signaling in MCF-7 cells.

We have previously demonstrated that H3K18ac can be used to detect stimuli specific co-activation (EGF vs. E2) at regulatory regions in which ERα binding occurs under both treatment [15]. Interestingly PBX1 depletion directly suppressed H3K18ac signal at several putative elements normally recruiting ERα (Fig. 3C). Globally, PBX1-EGF-ERα regions are characterized by open chromatin status as demonstrated by FAIRE-seq and DNaseI-hypersensitivity-seq assays (Fig. 3D). However, PBX1 depletion results in a significant decrease in chromatin openness (as demonstrated by reduced FAIRE signal) at several regions associated with EGF-ERα target genes suggesting that PBX1 modulates ERα signaling by influencing the chromatin environment.

### PBX1 is a novel luminal breast cancer prognostic biomarker

Our mechanistic study revealed that PBX1 controls the expression of a subset of ERα target genes stimulated by EGF *in vitro* and may be linked to breast cancer progression. Furthermore we previously demonstrated that PBX1 displays an analogous function by controlling a subset of estrogen induced genes strongly associated with poor prognosis [16]. In light of these observations we hypothesized that PBX1 expression levels could contribute to breast cancer progression in ERα-positive breast cancer patients. To test this hypothesis we performed meta-analysis on METABRIC (Illumina arrays) and The Cancer Genome Atlas (TCGA, RNA-seq data) studies stratifying ERα-positive breast cancer patients in high and low PBX1 expressors. In both datasets high PBX1 mRNA levels correlate with shorter survival (Fig. S4A). For the METABRIC dataset, PBX1 expression retained significance in a multivariate analysis including lymph node status, grade and tumor size (HR = 1.27, *p* = 0.0173). For the TCGA datasets the TNM status was published for each patient. In the multivariate analysis including PBX1, T, N and M, PBX1 reached the highest significance (*p* = 0.041, HR = 7.82), M also reached significance (*p* = 0.045, HR = 2.89) while T and N were not significant. These correlations were not significant in ERα-negative breast cancer patients further supporting the notion that PBX1 is a key regulator of ERα activity (Fig. S4A). Notably, higher PBX1 levels were significantly associated with poor prognosis in a subset of patients treated with endocrine therapy (Fig. S4B). Again, PBX1 retained significance in a multivariate analysis including lymph node status, grade, tumor size and PBX1 expression (HR = 1.27, *p* = 0.0173). To elucidate further the potential role of PBX1 as a breast cancer biomarker, we tested the association between its expression and metastatic progression in an additional six independent studies (Affymetrix arrays). Overall, PBX1 expression was associated with increased risk in metastatic progression (HR 1.47, *p* < 0.008, *n* = 674). We found more variability within the individual cohorts, however, statistical significance was reached only for datasets in which PBX1 represents a poor prognostic marker (Fig. S5). Additionally, multivariate analysis in the grouped cohort indicates that PBX1 prognostic power is independent from Ki67 (*p* < 0.0001)

**Figure 4:**
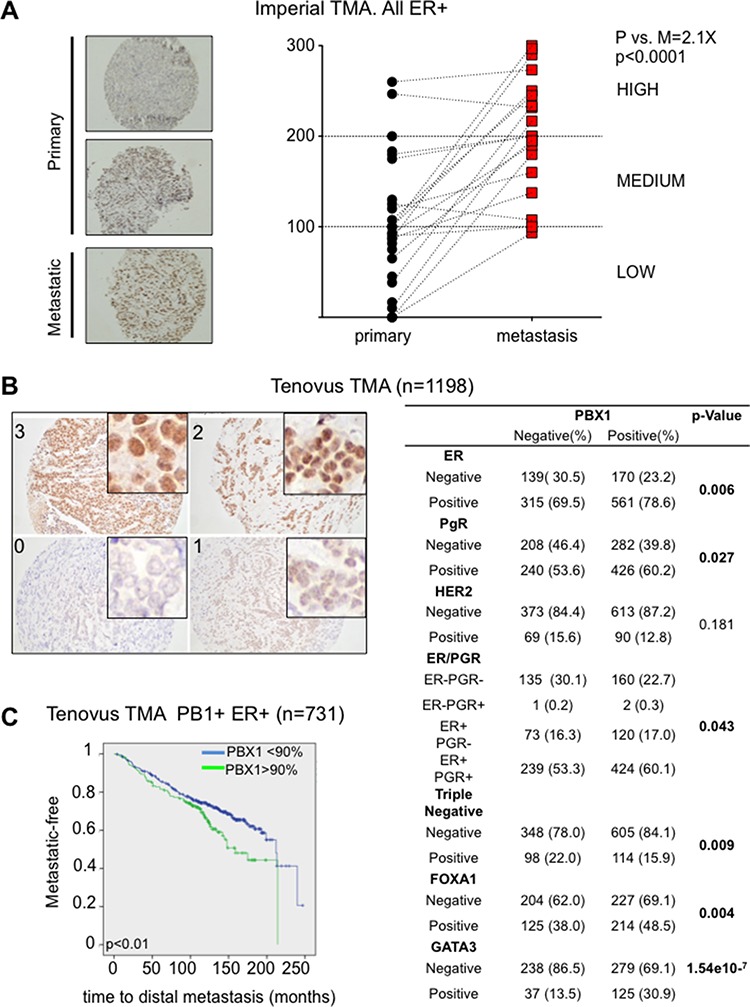
PBX1 is a novel prognostic biomarker for ERα breast cancer **A.** Primary and matched Metastatic samples from the Imperial College TMA were processed using PBX1 IHC and H score were plotted. Pair-wise *t*-test between the average score (3 independent scorer, duplicate sections) was used to establish significance **B.** Core primary biopsies in the Tenovus Nottingham cohort show different level of staining by PBX1 IHC. PBX1 correlates with several important clinico-pathological parameters including hormone status and ERα associated pioneer factors. **C.** Survival analysis (Log-rank Mantel-Cox test) was performed looking at patients with very high PBX1 levels versus the remaining patients. Analysis was restricted to the to the ERα-positive population.

To confirm these findings we measured PBX1 protein levels using IHC in longitudinal clinical samples. We collected 20 biopsies from primary ERα-positive breast cancers and compared PBX1 protein levels with matched relapses (all endocrine treated, average time to relapse = 44.4 ± 35 months). We stained all sections using a validated antibody (Fig. S1C and S2A) [5, 16] and scored each sample based on PBX1 nuclear intensity (Fig. 4A). Remarkably, metastatic samples had a significant increase in H score compared to the original primary biopsies confirming PBX1 upregulation in drug-resistant metastatic patients (Fig. 4A). Finally, we investigated PBX1 protein levels in the Tenovus Nottingham cohort of FFPE primary breast cancers (*n* = 1650) [23]. In agreement with cell line data, we found that PBX1 protein levels significantly correlate with ERα, FOXA1 and GATA3 (another important ERα pioneer factor [22]) thus demonstrating that PBX1 is strongly associated with ERα-positive, luminal breast cancer subtypes (Fig. 4B). We therefore restricted further analysis to ERα-positive breast cancer patients. Since PBX1 protein levels were dramatically increased in metastatic samples, we hypothesized that high levels of PBX1 in primary samples might identify patient at higher risk of relapse. Our results show that ERα-positive breast cancer patients with elevated PBX1 staining intensity at diagnosis (over 90% positive staining) develop metastatic disease significantly faster than patients with lower staining intensity (Fig. 4C). Overall this data strongly suggest that PBX1 protein levels in primary breast cancer are significantly associated with breast cancer progression.

### PBX1 locus is amplified in patients with aggressive ERα breast cancer

Our analysis using PBX1 transcriptional and protein levels strongly supports the role of PBX1 as a functional biomarker associated with ERα breast cancer progression (Figs. 4 and S4–S5). PBX1 transcripts are significantly overexpressed in breast cancer when compared to normal tissues (Fig. S6). For example, PBX1 ranks in the top 50 most overexpressed genes in ductal and lobular carcinoma compared to normal tissue in TCGA cohort (dataset 30 in Fig. S6) [24]. Similarly, PBX1 ranks in the top 4% of the most overexpressed transcripts in the METABRIC cohort (Invasive Ductal vs. Normal, dataset 7, Fig. S6) [25]. To the best of our knowledge, there is no indication in the literature that PBX1 is required for normal breast development. In agreement, we found that normal ductal cells adjacent to tumor tissue have very low PBX1 staining (stronger stained cells are myoepithelial cells, Fig. S7). Thus, PBX1 expression seems to be a major determinant for the development and progression of luminal breast cancer patients.

Recent tumour sequencing efforts indicate that PBX1 locus is potentially amplified in 13% of primary breast cancers (TCGA provisional) (Fig. S8A) [26] supporting the hypothesis that genomic amplification may partially explain PBX1 overexpression in breast cancer (TCGA provisional) (Fig. S8B). Of note, within the published TCGA dataset [24], PBX1 potential amplification strongly correlates with higher mRNA levels in ERα-positive but not in ERα-negative breast cancer patients (Fig. S9). These data suggest that PBX1 copy number variation (CNV) might then be functional only in ERα-positive breast cancer patients. We then identified the TCGA patients (TCGA provisional) with ERα-positive disease that had at least 3 copies of PBX1 (95/1145, 8.3%) and analysed the proportion of patients with lymph-node micro-metastasis. Strikingly, we found that 60% of patients with PBX1 amplification had at least one positive lymphnodes vs. 38.7% of the control group (Fisher exact test 7.3* 10^−5^). Conversely, PBX1 amplification correlates, although not significantly, with a lower chance of metastatic disease in the limphnodes in ERα negative breast cancer patients (45% vs. 57% respectively).

**Figure 5:**
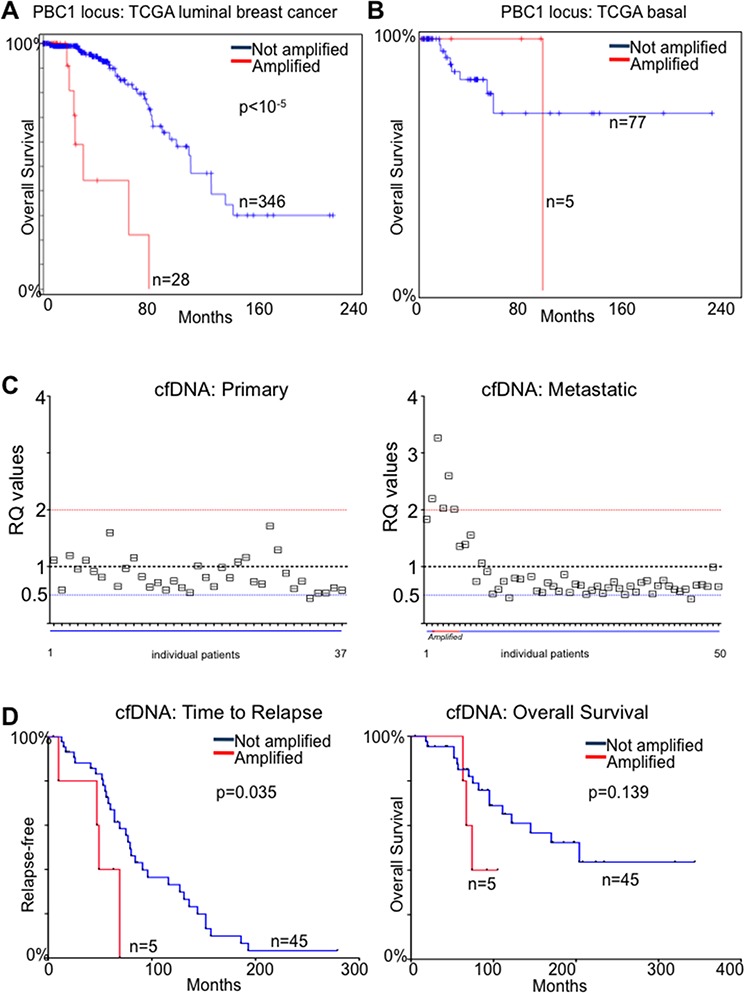
PBX1 amplification is a prognostic biomarker for ERα breast cancer **A.** TCGA luminal patients [24] were stratified based on putative PBX1 amplification and overall survival was calculated **B.** The same analysis was repeated in basal and claudin low patients **C.** qPCR measurement of PBX1 genomic DNA in cfDNA from primary and metastatic ERα breast cancer patients **E–F.** Metastatic patients from panel C were stratified based on PBX1 amplification and Time to relapse and Overall survival were calculated. Groups were compared using a Log-rank Mantel-Cox test.

We then restricted our analysis to ERα-positive breast cancer patients from the published TCGA data to exploit the more complete clinical annotations [24] and found that 28/364 (8%) carried potential PBX1 amplification/overexpression (Fig. 5A). Patients with putative PBX1 amplification had a median survival of 30.98 months compared to 113.74 (PBX1 Amplified vs. Not Amplified in Luminal patients, *P* < 10^−5^) (5A). On the other hand, PBX1 amplification did not reach prognostic significance in ERα negative patients (however, only 6% of patients carries amplification) (Fig. 5B). When we looked at genes potentially co-amplified with PBX1 (chr1q23.3) [27] we found that very few candidates were also over-expressed in breast cancer (Fig. S10A). More importantly, PBX1 was one of the only two transcripts (the other being PVRL4) carrying evidence of prognostic significance in the ERα-positive breast cancer subtype (METABRIC) (Fig. S10B).

We then considered the possibility of using PBX1 CNV as a proxy for PBX1 protein levels. Potentially, this would allow for monitoring PBX1 levels by measuring its amplification in DNA derived from cancer cells using non-invasive methods. The tumour derived fraction of circulating free-DNA (cfDNA) originating from tumour cells can be used to estimate the genomic status of breast cancer lesions [28–30]. We then measured PBX1 amplification using a qPCR assay in cfDNA from 37 patients with ERα-positive primary disease and 50 patients with ERα-positive metastatic disease treated with endocrine therapy (Table S2). We could not identify significant CNV in blood from primaries ERα-positive breast cancer patients. However, we identified 5/50 (10%) patients with PBX1 amplification (RQ > 2) and two patients with probable allelic loss (4%) in the metastatic cohort (Fig. 5C). We then reasoned that the emergence of PBX1 CNV may have contributed to tumor progression and used PBX1 amplification in cfDNA as a classifier to stratify patients based on time to relapse or overall survival. In this small cohort, PBX1 amplification was significantly associated with earlier metastatic relapse (*p* < 0.035, HR 5.95, Cox- Mantel-Test) (Fig. 5D). These data are in strong agreement with our protein and transcriptional tumour profiling and coupled with our mechanistic study demonstrate that PBX1 plays a crucial role in ERα-positive breast cancer development and progression.

## DISCUSSION

Our work has identified PBX1 as a novel functional biomarker in ERα-positive breast cancer. We have identified a mechanistic association between PBX1 and ERα recruitment in response to EGF signaling. We also established the significance of PBX1 mRNA and protein level as a prognostic biomarker in several independent large cohorts of primary breast cancers. Notably, PBX1 levels are strongly associated with metastatic progression. Finally, we demonstrate that the longitudinal increment of PBX1 levels observed throughout cancer progression are partly due to genomic events that could be potentially used to monitor disease progression in patients by non-invasive assays.

The number of signaling molecules potentially involved in breast cancer is rapidly increasing. In this context, ERα remains a central hub required to regulate and integrate signaling at the chromatin level [31, 32]. EGF signaling cascade partially converge on ERα in breast cancer cells where it provides for an additional survival pathways. ERα target genes induced via classical estrogen signaling and non-canonical EGF signalling are quite distinct [14, 15]. Nonetheless, PBX1 remains a common denominator of these two pathways via its conserved interaction with ERα. It will be interesting to investigate the role of pioneers factors in alternative ERα pathways, including metabolic induced ERα signaling [33, 34]

Genetic aberration targeting chromatin remodelers and other proteins involved in epigenetic processes are now frequently recognized as major drivers in several types of cancers [35]. Synergy between epigenetic and genetic drivers may be particularly important in breast cancer considering the essential role of ERα in these malignancies and the intimate crosstalk between ERα and the chromatin [21, 22, 36]. For example, it is possible that PBX1 overexpression may lead to an increased number of chromatin accessible sites thereby allowing ERα binding near oncogenes [37]. While several other pioneer factors play a central role in modulating ERα binding in breast cancer cells [16, 22, 36, 38, 39], our data suggest that PBX1 has some unique properties. For example, FOXA1 [39] and GATA3 [40] play a central role in mammary and ductal morphogenesis and their interaction with ERα can be regarded as developmentally conserved [41, 42]. On the other hand PBX1 expression is restricted to ERα transformed cells and practically absent in normal ductal cells. In addition, FOXA1 and GATA3 are frequently targeted by mutations in breast cancer [43] (Fig. S11), while PBX1 is rarely affected by somatic mutations (V117M and H425N, 2 out of 892 patients). Furthermore FOXA1 and GATA3 are frequently downregulated in basal tumour but rarely over-expressed or amplified in luminal cancer (Fig. S11) [44] while PBX1 is among the top upregulated genes and is frequently amplified in luminal cancers (Figs. 4–5, S6, S8, S10 and S11).

Despite the impossibility to elucidate the temporal details of PBX1 amplification/overexpression, our data strongly suggest that PBX1 upregulation may contribute to breast cancer progression and metastatic development. Clonal evolution is thought to contribute significantly to cancer progression [45]. In this context, we can envision two possible models: in the first, cells carrying PBX1 amplification may be more adapted to escape the primary site and colonize distal tissues. On the other hand, it is possible that endocrine therapies exert positive selective pressure on cells that incur in Chr1q23.3 gains. In both scenarios, it is tempting to speculate that PBX1 would contribute to epigenetic reprogramming by interpreting or shaping the histone modifications landscape [21]. Ultimately, these changes would modulate ERα signaling and allow breast cancer cells to have an increased fitness in response to a diverse array of signalling pathways.

## MATERIALS AND METHODS

### Cell lines

MCF-7 and T47D cells were cultured and maintained as previously described [16]. EGF stimulation was performed as previously described [15]. Proliferation curves were performed as previously described [16]. Treatment with GSI (10uM of MRK003 or PF03084014) was performed as previously described [5]. Experiments were repeated independently at least five times.

### RT-qPCR and microarray

RNA samples from siControl or siPBX1 treated MCF7 in the presence or absence of EGF were extracted with RNAeasy (Quiagen) and retro-transcribed with iSCRIPT (Biorad). For microarrays, RNAs were hybridized on HT12 human beads array (Illumina Inc.). Analyses were performed using BRB- Array Tools Version 3.8.1. Raw intensity data were log2 transformed, median normalized and filtered to remove non- detected spots as determined by Illumina Software. The normalization was performed by computing a gene-by-gene difference between each array and the median (e.g. reference) array, and subtracting the median difference from the log intensities on that array, so that the gene-by-gene difference between the normalized array and the reference array is zero. Two class non-paired comparison analyses were performed by computing a *t*-test for each gene using normalized log-intensities. Differentially expressed genes were determined at a significance level of q less than 0.01. A four class ANOVA at p less than 0.01 was also performed to identify genes expressed differentially across the four groups. Hierarchical clustering was employed by calculating Euclidean distance to generate heat maps for subsets of significant genes using the open source software Cluster/Treeview.

### ChIP and ChIP-seq

ChIP assay were conducted as described previously described using PBX1 (Abnova, M01 clone 4A2) or ERα (Santa Cruz biotechnology) [16] using 5–10* 10^6^ cells. Library preparation for next-generation sequencing was performed according to manufacturer's instruction starting with 5 ng of material (Illumina Inc.). Single paired libraries were sequenced using the HT2500 (Illumina Inc). Over 20 million 50 bp reads were generated through for the ERα ChIP and Input samples. Of those, over 92% were aligned to the human reference genome. These reads were aligned using bowtie [46] (default setting). The MACS peak-calling algorithm was used to call significantly enriched peaks using default settings (*P* < 10–5) [47].

### Ontology analysis and heatmap analysis

Ontology analyses were performed using GREAT [48]. Heatmaps were generated using CHASE [49].

### Imperial tissue microarray of paired primary and secondary breast cancers

Twenty primary breast carcinomas with a paired metastasis were acquired from the pathology archives of Charing Cross Hospital, London, UK. A tissue microarray was constructed using a manual microarrayer (Beecher) and 0.6 mm punches. The tissue microarray was immunohistochemically profiled for PBX1 and other biomarkers as previously described [16]. PBX1 monoclonal antibody (Abnova, (M01), clone 4A2) was optimized to a working concentration using tissue sections (5 μm). Antigen retrieval was performed using 10mM Tris-HCl with 1 mM EDTA (pH 9.0), heated in a water bath at 95°C for 30 min followed by cooling at room temp for 30 min. Blocking was done using 0.3% hydrogen peroxide in PBS, followed by normal goat serum (20 μl per ml) for 30 min. The primary antibody (1:50 in PBS) was incubated overnight at 4°C, then detected using anti-rabbit secondary antibody, Vectastain Elite peroxidase ABC kit, and ImmPACT DAB kit (all from Vector Laboratories). Subsequently, immunohistochemistry was performed on TMA sections (4–5 μm) using the optimized staining protocol, including negative controls (omission of the primary antibody). PBX1 immunoreactivity was localized in the nucleus. Staining was scored based on the H-score and Allred Quick score (LM, DKP and PV).

### Nottingham tenovus primary breast cancer series

Primary operable breast cancer cases (*n* = 1650) from the Nottingham Tenovus Primary Breast Carcinoma Series were used and were utilized for immunohistochemistry. Clinical data were maintained on a prospective basis with a median follow-up of 126 months [23, 50]. The tissue microarrays and full-face sections form the Nottingham Tenovus Primary breast cancer series were immunohistochemically profiled for PBX1 and other biological antibodies as previously described. PBX1 mouse monoclonal antibody (Abnova, (M01), clone 4A2 [16] was optimized to a working concentration, utilizing μm matched full- face excisional tissue sections. Antigen retrieval was performed using Leica ER2 (pH 9.0) retrieval solution, water bath at 95C for 35 minutes followed by TBS at 50C for 10minutes. Blocking was done using Thermo Fisher UltraV block 5mins. Antibody concentration was 1:50 overnight at 4°C. Subsequently, 4 μm TMA sections were immuno-stained using the optimized staining protocol. Detection was achieved using the Novalink Polymer Detection kit (Leica Micro- systems Inc., USA). Negative controls were performed by omission of the primary antibody. IHC revealed that PBX1 had a nuclear location (4B). Nuclear staining was scored based on the H-score and Allred Quick score. Determination of the optimal cut-offs was per- formed using histograms and confirmed using X-tile bio-informatics software (Yale University, USA) [51] where data were split into training and validation sets. A total of 1198 tumours were suitable for analysis (460 PBX1 negative and 738 PBX1 positive). The tumour cores were evaluated by two of the coauthors (DKP and PV) blinded to the clinico-pathological characteristics of patients. There was substantial intra- and inter- observer agreement (k [0.768; Cohen's j and multi-rater j tests, respectively).

### Survival analysis using transcriptomics data

Positive PBX1 expression was dichotomised to 90% using distant metastases as an outcome and determined through X-tile software (University of Yale, Yale, USA) [51]. Prognostic analyses were undertaken using Kaplan-Meier curves using the Log-rank test. A *p*-value < 0.05 was considered significant.

### Survival analysis using transcriptomics data

Database construction and survival analysis was performed as described previously [52]. For the expression of the genes, the median expression was used as the cut-off in a Cox regression analysis. Kaplan-Meier survival plot, and hazard ratio with 95% confidence intervals and logrank *P* value were calculated and plotted in R using Bioconductor packages.

### Construction of METABRIC microarray database

The Metabric project employed Illumina microarrays to measure gene expression across all genes. The raw gene chip files were downloaded from the European Genome-phenome Archive (EGA) (https://www.ebi.ac.uk/ega/) [25]. The entire cohort contains 1988 samples, the average overall survival is 8.07 years, 76% of the samples are ER positive and 47.3% are node positive. Due to batch effects between the Metabric training and validation sets we have not used the pre-normalized tables but have re-run the complete pre-processing for all arrays. In this, raw data was first imported into R and summarized using the beadarray package [53]. For annotation, the Illumina Humanv3 database of Bioconductor was used (http://www.bioconductor.org). Finally, quantile normalization was performed using the preprocessCore package [54].

### FAIRE and DNaseI-hypersensitivity-seq

FAIRE-seq and DHS-seq analysis were performed as previously described [16, 55] with minor modifications.

### Circulating free DNA analysis

DNA was extracted from blood cell pellets and 1ml plasma as described previously [30]. To confirm amplification of PBX1, 10ng cfDNA was subjected to 5 cycles of preamplifcation with a mix of primers (including PBZ1 and reference genes). Each sample was then analysed in triplicate by real-time quantitative PCR (qPCR) in a 10 μl reaction volume. Reactions were run on an Applied Biosystems thermal cycler (Step One Plus) and analysed with Step One v2.1 software and Microsoft Excel.

### Primers

Primers sequences (RT-qPCR, ChIP-qPCR) are available upon requests. Primers to study PBX1 amplification were as follow: Primers PBX1-F: AGCCCACTCATCTTACGTGAC PBX1-R: ACGAAATTCCACTCCAACTCCA Probe PBX1 GCTCAGGCCTATCTTCTGGA FAM-MGB.

## SUPPLEMENTARY MATERIALS







## Abbreviations

ERα: Estrogen Receptor α
EGF: Epidermal Growth Factor
TF: Transcription factor
cfDNA: circulating free-DNA
FAIRE: Formaldehyde assisted isolation of regulatory elements
CNV: copy number variation
