# Supplementary material for: The pioneer factor PBX1 is a novel driver of metastatic progression in ERα-positive breast cancer
Source: Oncotarget. 2015 Jun 15;6(26):21878–91. doi: 10.18632/oncotarget.4243 (PMC4673133; doi:10.18632/oncotarget.4243)
Supplement: Supplementary file 1 [file oncotarget-06-21878-s001.pdf]

SUPPLEMENTARY MATERIALS

REFERENCES

1. Rhodes DR, Kalyana-Sundaram S, Mahavisno V, Barrette TR, Ghosh D, Chinnaiyan AM. Mining for regulatory programs in the cancer transcriptome. Nat Genet. 2005; 37:579–83.
2. Cerami E, Gao J, Dogrusoz U, Gross BE, Sumer SO, Aksoy BA, Jacobsen A, Byrne CJ, Heuer ML, Larsson E, Antipin Y, Reva B, Goldberg AP, Sander C, Schultz N. The cBio cancer genomics portal: an open platform for exploring multidimensional cancer genomics data. Cancer Discov. 2012; 2:401–4.
3. Cancer Genome Atlas Network. Comprehensive molecular portraits of human breast tumours. Nature. 2012; 490:61–70.

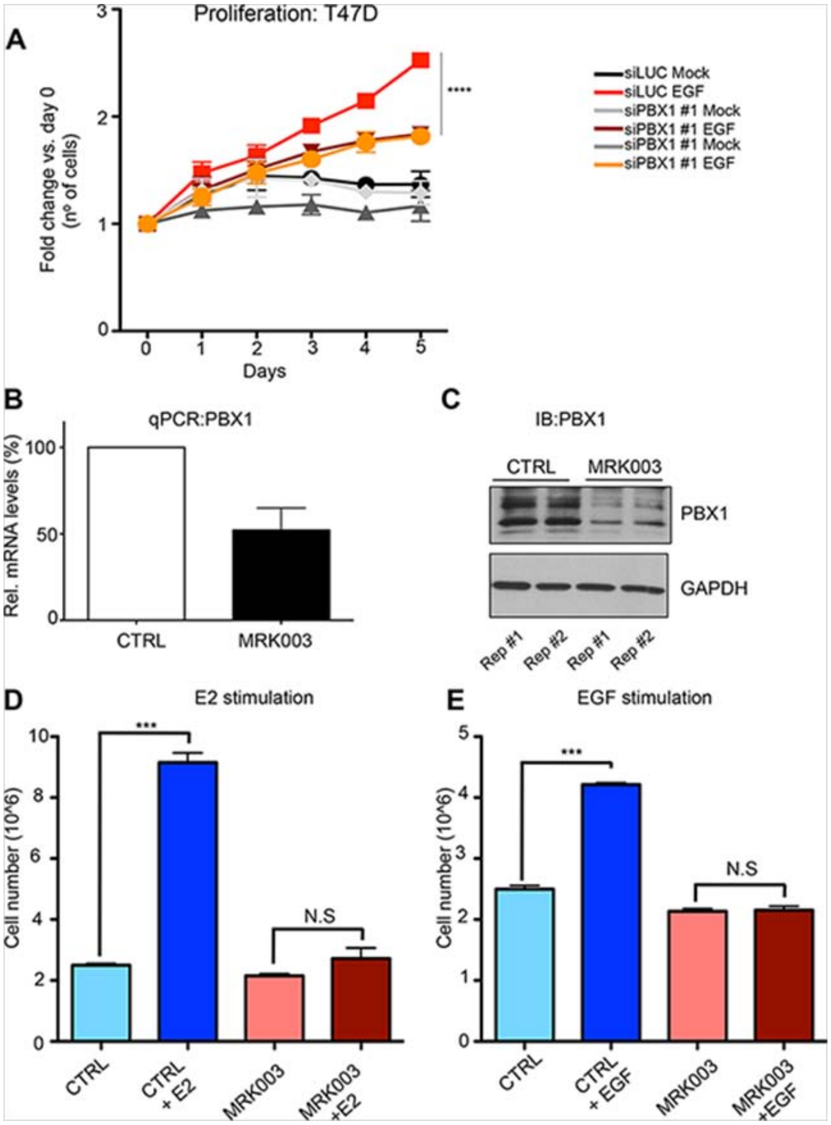

Supplementary Figure S1: A. Proliferation curve of ERα T47D breast cancer cells in response to EGF stimulation (100 ng/mL) treated with siRNA against PBX1. B. PBX1 mRNA and **C.** protein levels in MCF7 cells after treatment with GSI (10 uM) **D.** MCF7 proliferation response to estradiol treatment in presence or absence of GSI treatment (10 uM) **E.** MCF7 proliferation response to EGF treatment in presence or absence of GSI treatment (10 uM). Asterisks identify significant differences between treatments.

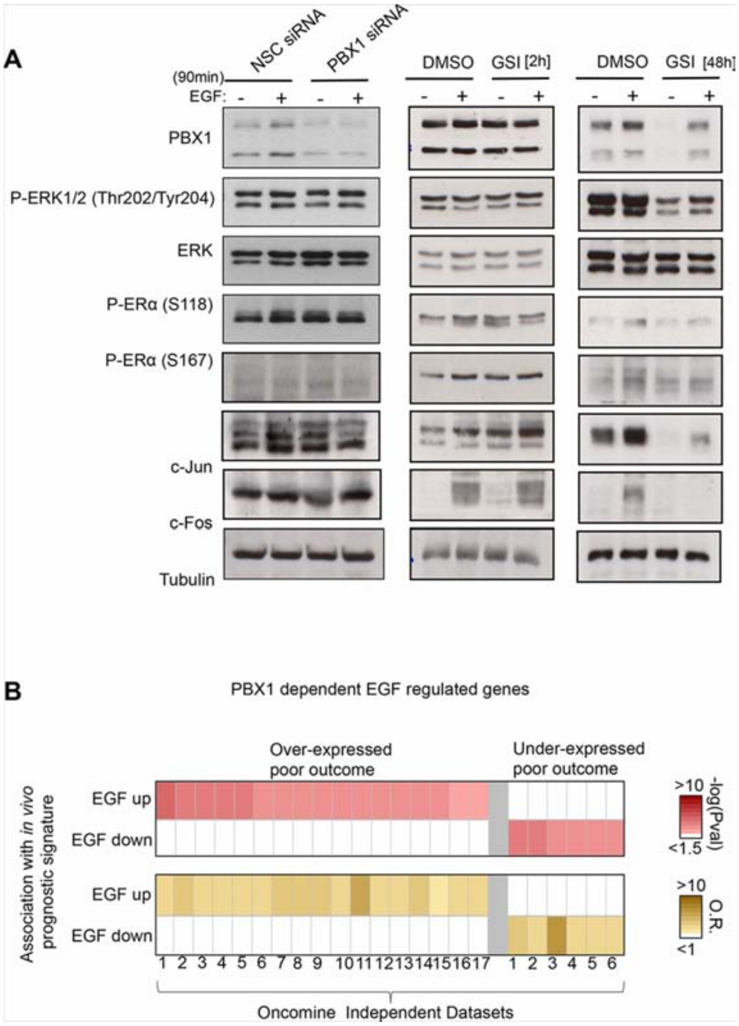

datasets	-LOG(pVAL)	
Invasive Ductal Breast Carcinoma - Recurrence at 3 Years - Top 10% Over-expressed (Desmedt Breast)	4.92811799	1
Ductal Breast Carcinoma - Recurrence at 3 Years - Top 5% Over-expressed (Sorlie Breast)	3.55284197	1
Breast Carcinoma - Recurrence at 3 Years - Top 10% Over-expressed (vandeVijver Breast)	3.49349497	1
Breast Carcinoma - Recurrence at 3 Years - Top 10% Over-expressed (Loi Breast 3)	3.19586057	1
Invasive Ductal Breast Carcinoma - Metastatic Event at 3 Years - Top 10% Over-expressed (Desmedt Breast)	3.11747546	1
Breast Carcinoma - Dead at 3 Years - Top 10% Over-expressed (vandeVijver Breast)	3.09963287	1
Ductal Breast Carcinoma - Dead at 5 Years - Top 5% Over-expressed (Sorlie Breast)	3	1
Ductal Breast Carcinoma - Recurrence at 1 Year - Top 5% Over-expressed (Sorlie Breast)	3	1
Ductal Breast Carcinoma - Dead at 5 Years - Top 5% Over-expressed (Sorlie Breast 2)	2.52287875	1
Breast Carcinoma - Dead at 5 Years - Top 10% Over-expressed (Kao Breast)	2.52287875	1
Breast Carcinoma - Recurrence at 1 Year - Top 1% Over-expressed (Wang Breast)	2.52287875	1
Breast Carcinoma - Dead at 5 Years - Top 10% Over-expressed (vandeVijver Breast)	2.39794001	1
Ductal Breast Carcinoma - Recurrence at 1 Year - Top 10% Over-expressed (Sorlie Breast 2)	2.22184875	1
Breast Carcinoma - Dead at 3 Years - Top 1% Over-expressed (Kao Breast)	2.04575749	1
Breast Carcinoma - Metastatic Event at 3 Years - Top 10% Over-expressed (vandeVijver Breast)	2.04575749	1
Invasive Breast Carcinoma - Metastatic Event at 1 Year - Top 10% Over-expressed (Hatzis Breast)	2	1
Invasive Ductal Breast Carcinoma - Metastatic Event at 1 Year - Top 10% Over-expressed (Desmedt Breast)	2	1
Breast Carcinoma - Metastatic Event at 1 Year - Top 5% Under-expressed (Kao Breast)	1	3.43415218
Ductal Breast Carcinoma Epithelia - Dead at 1 Year - Top 10% Under-expressed (Boersma Breast)	1	3.2873503
Invasive Breast Carcinoma - Metastatic Event at 3 Years - Top 1% Under-expressed (Hatzis Breast)	1	2.39794001
Breast Carcinoma - Metastatic Event at 3 Years - Top 10% Under-expressed (Minn Breast 2)	1	2.39794001
Breast Carcinoma - Metastatic Event at 5 Years - Top 10% Under-expressed (Minn Breast 2)	1	2.39794001
Breast Carcinoma - Dead at 1 Year - Top 10% Under-expressed (Bild Breast)	1	2.04575749

Supplementary Figure S2: A. Western blot analysis of protein involved in the EGF signaling cascade in MCF7 cells depleted or not of PBX1. B. PBX1 dependent ERα-EGF target genes correlate with genes highly expressed in poor outcome breast cancer (17 independent datasets [1]). Genes repressed by PBX1-EGF-ERα associated are generally under-expressed in poor outcome breast cancer (6 independent datasets). Datasets information are reported below)

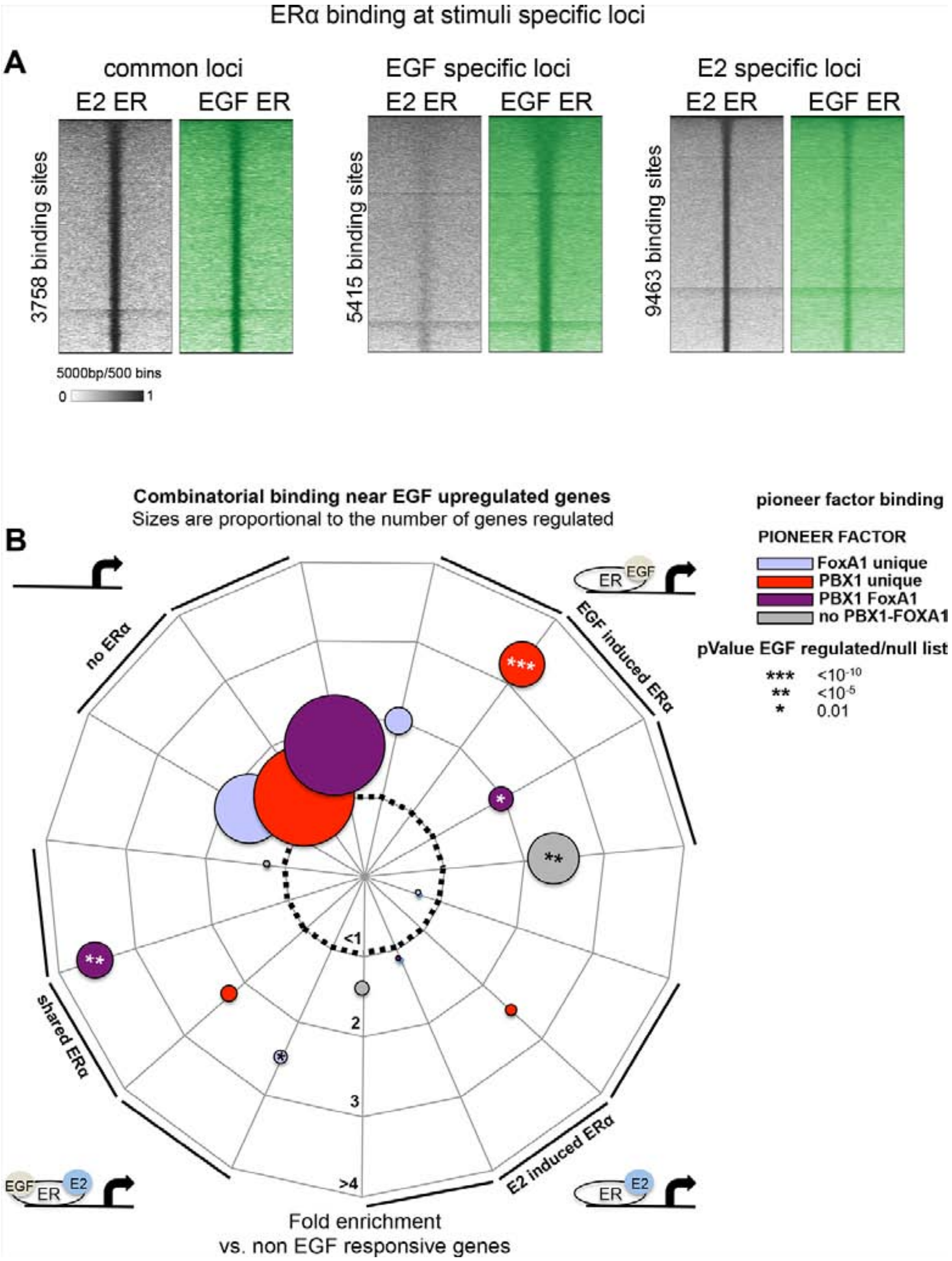

Supplementary Figure S3: A. Heatmaps showing raw ERα signals at genomic locations recruiting ERα in response to specific stimuli (EGF or E2) and loci recruiting ERα in response to both signalling molecules. B. Radar plot for ERα-pioneer factors interactions near EGF responsive genes. PBX1 dependent genes were identified and the genomic locations (−20 Kb from the TSS) were scanned for potential combinatorial binding. A similar analysis was performed for a transcriptional null-list (genes not activated upon EGF signaling). Numbers indicate fold increase (n° of genes with binding vs. n° of gene without binding near EGF/ n° of genes with binding vs. n° of gene without binding near null list) for each combinatorial binding.

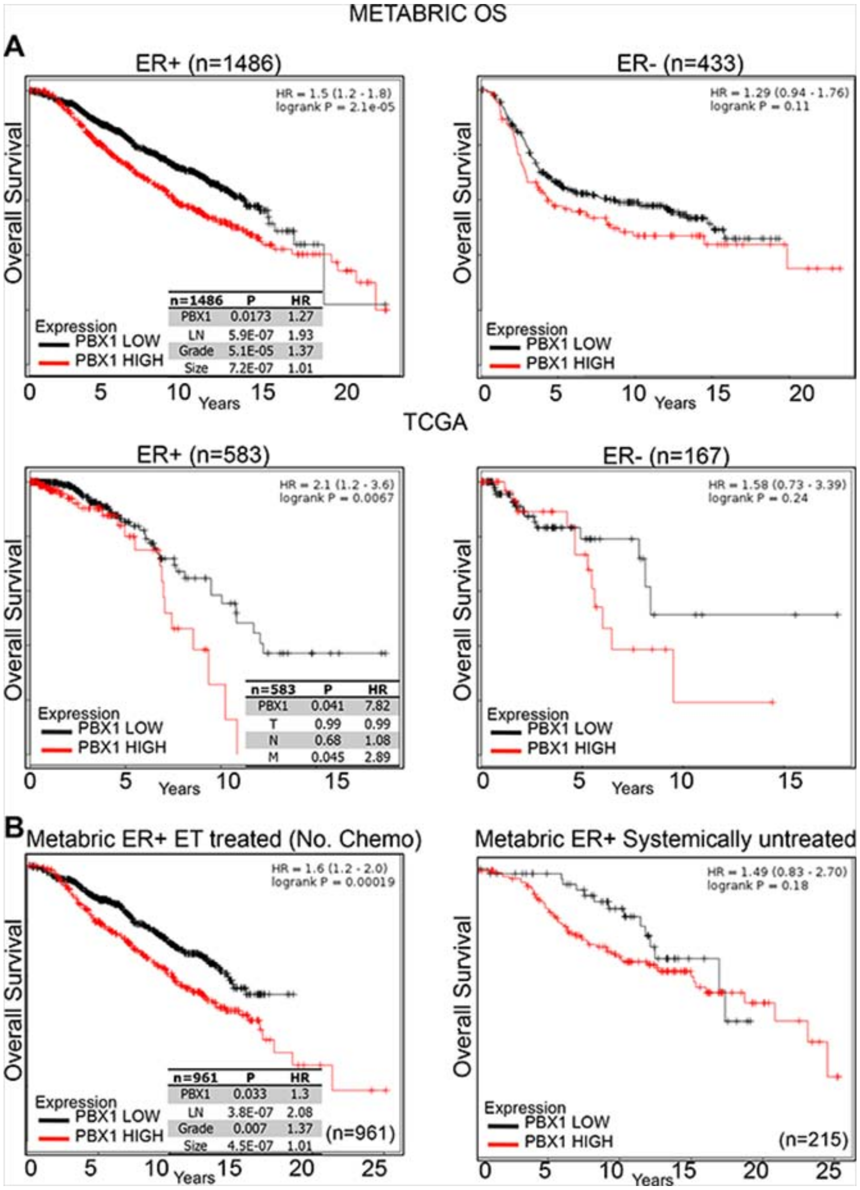

Supplementary Figure S4: A. Survival analysis using published available datasets using PBX1 expression (Illumina microarray or RNA-seq) as a classifier to discriminate outcome of patients with breast cancer. Analyses were conducted in ERα-positive and ERα-negative patients independently. Multivariate analysis is shown in the insets: T = size of the tumour, N = lymph nodes status, M = metastasis status **B.** Survival analysis using PBX1 transcripts levels were restricted to patients treated or not with endocrine therapies.

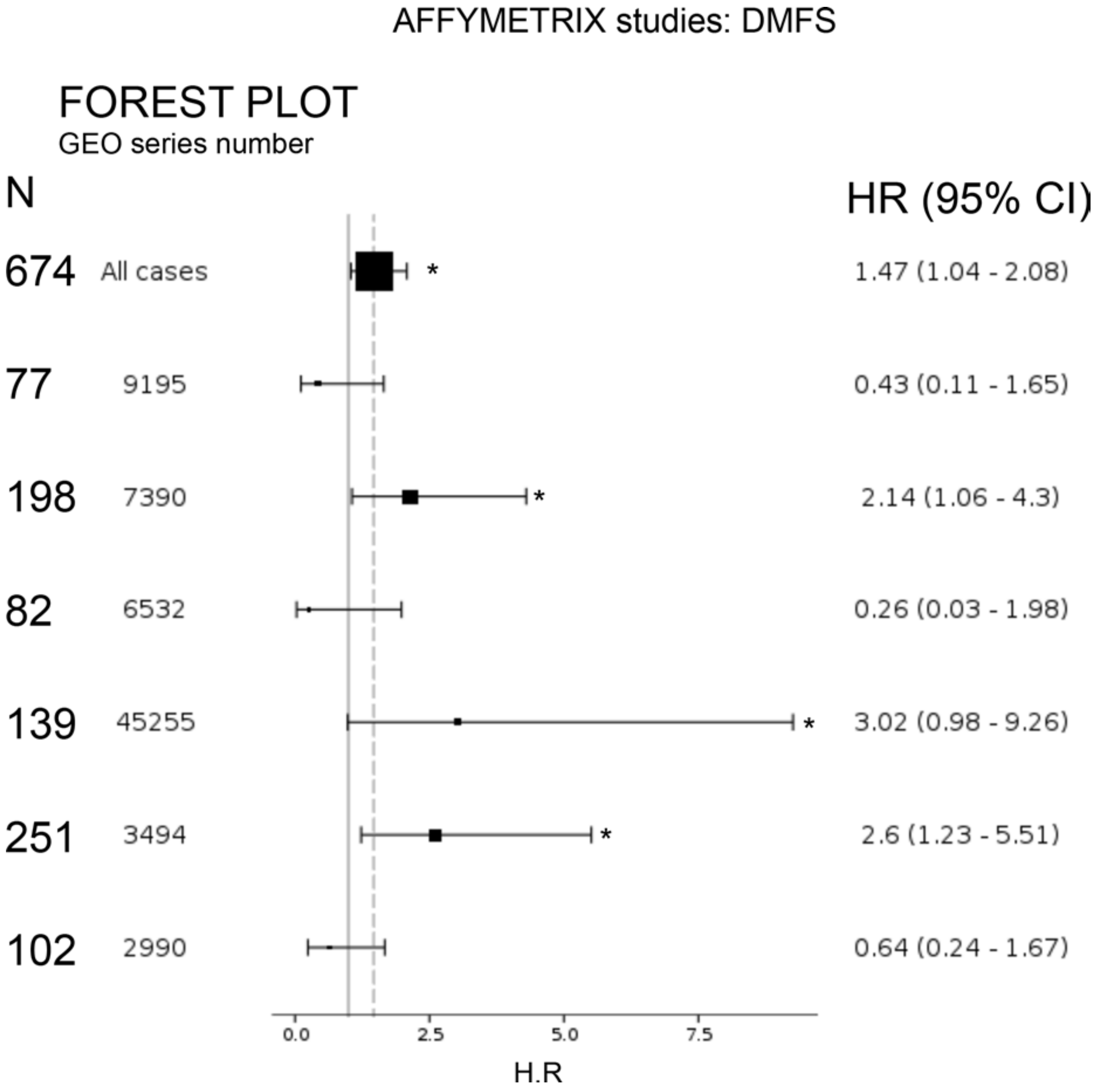

Supplementary Figure S5: Similar analyses to figure S4 were done using PBX1 transcript levels in six independent, previously published datasets using Affymetrix arrays (GSE9195, GSE7390, GSE6532, GSE45255, GSE3494 and GSE2990) as well as all these datasets combined ("All cases"). Hazard rates with 95% confidence intervals are shown. Size of the rectangles represents the sample number. Asterisks identify significant ($p < 0.05$) H.R values.

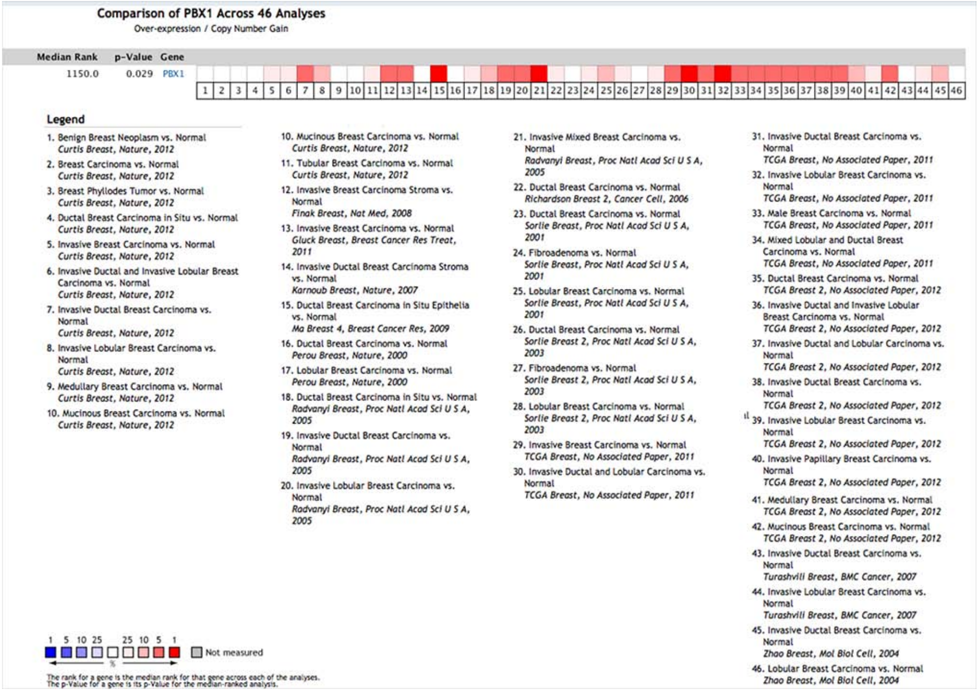

Supplementary Figure S6: Oncomine [1] datasets were used to compare PBX1 expression between normal breast and breast cancer samples. Datasets used in the analysis are listed in the figure.

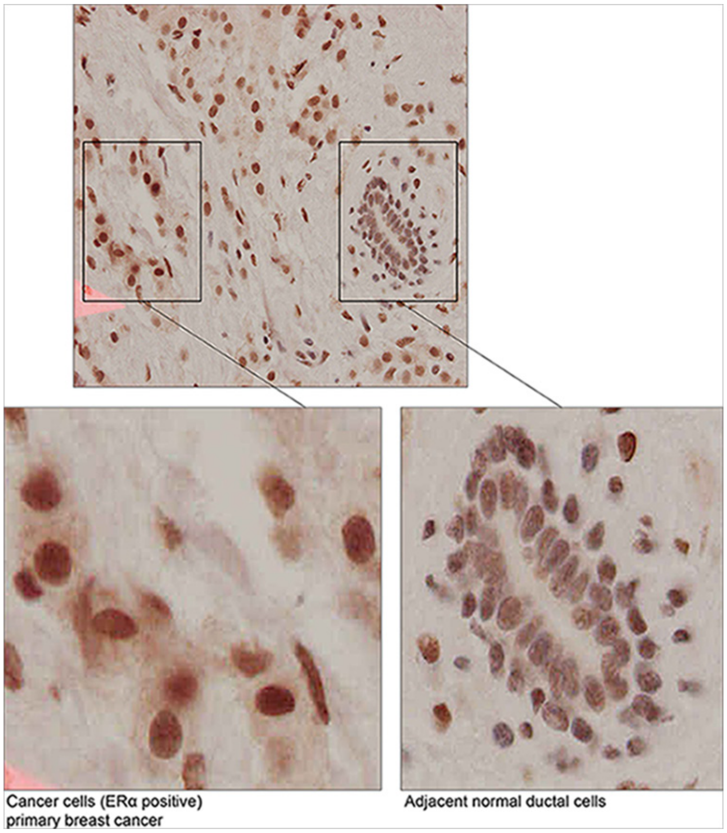

Supplementary Figure S7: PBX1 IHC staining in ductal cells from normal breast is shown.

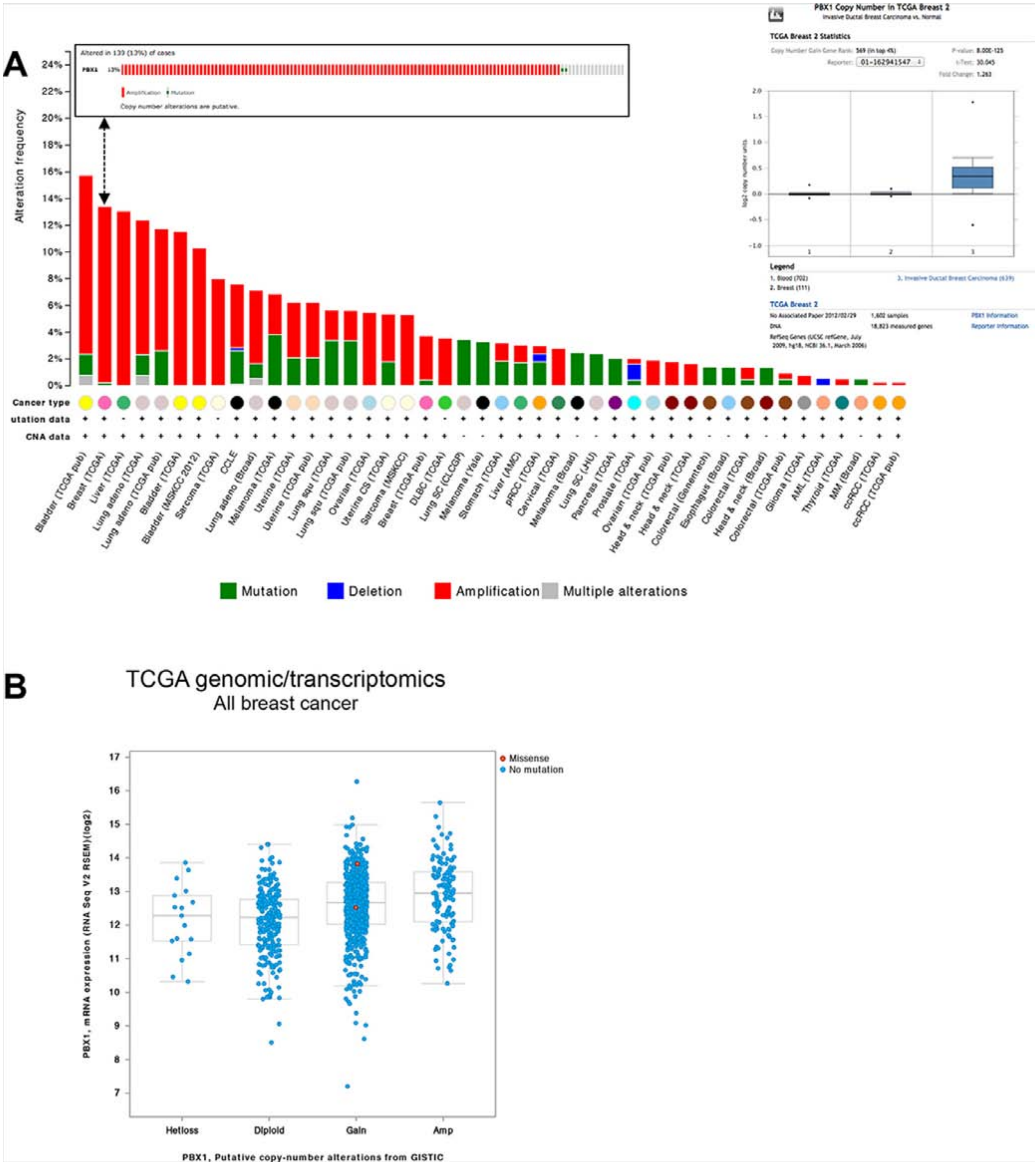

Supplementary Figure S8: A. Cbio portal summary analysis for PBX1 genomics aberration [2] in a large panel of cancers. Inset includes all provisional TCGA breast cancer data. Right panel shows copy number change in the TCGA panel [3]. PBX1 ranks in the top 4% most commonly amplified genes. **B.** TCGA (provisional) correlation between PBX1 CNA and transcriptional levels.

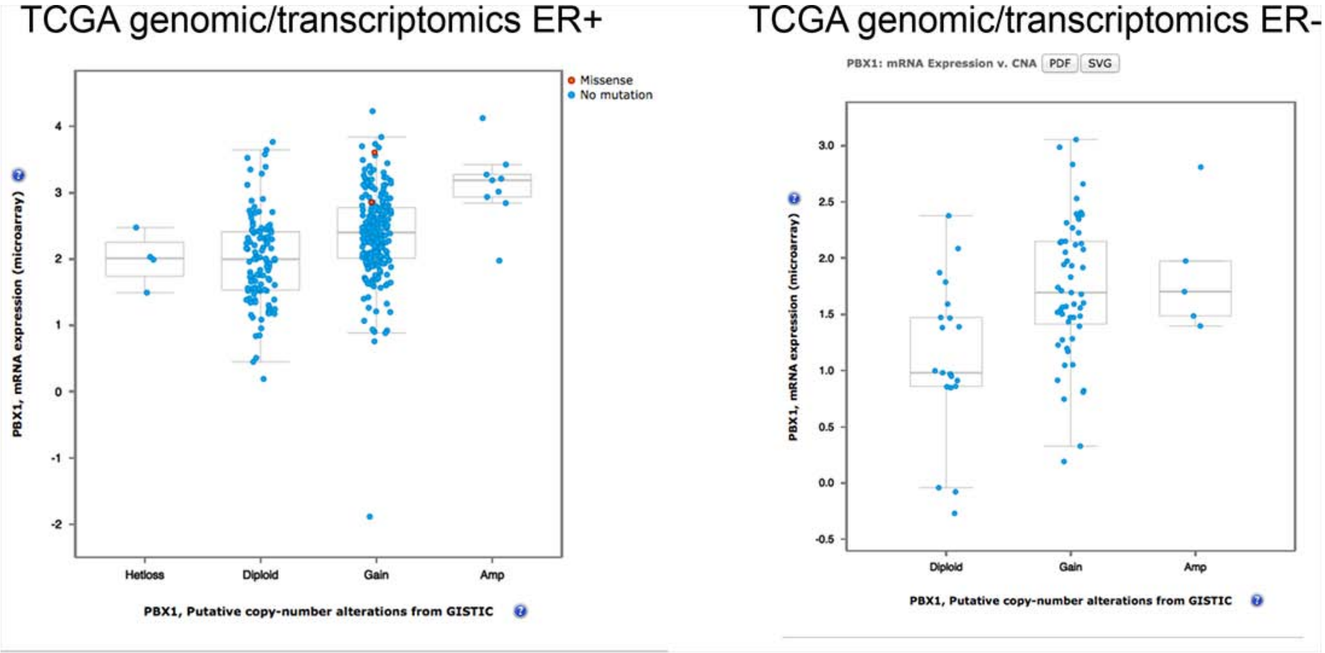

Supplementary Figure S9: TCGA [3] correlation between PBX1 CNA and transcriptional levels in ERα-positive and ERα-negative breast cancers. Analyses were conducted using the Cbio portal [2].

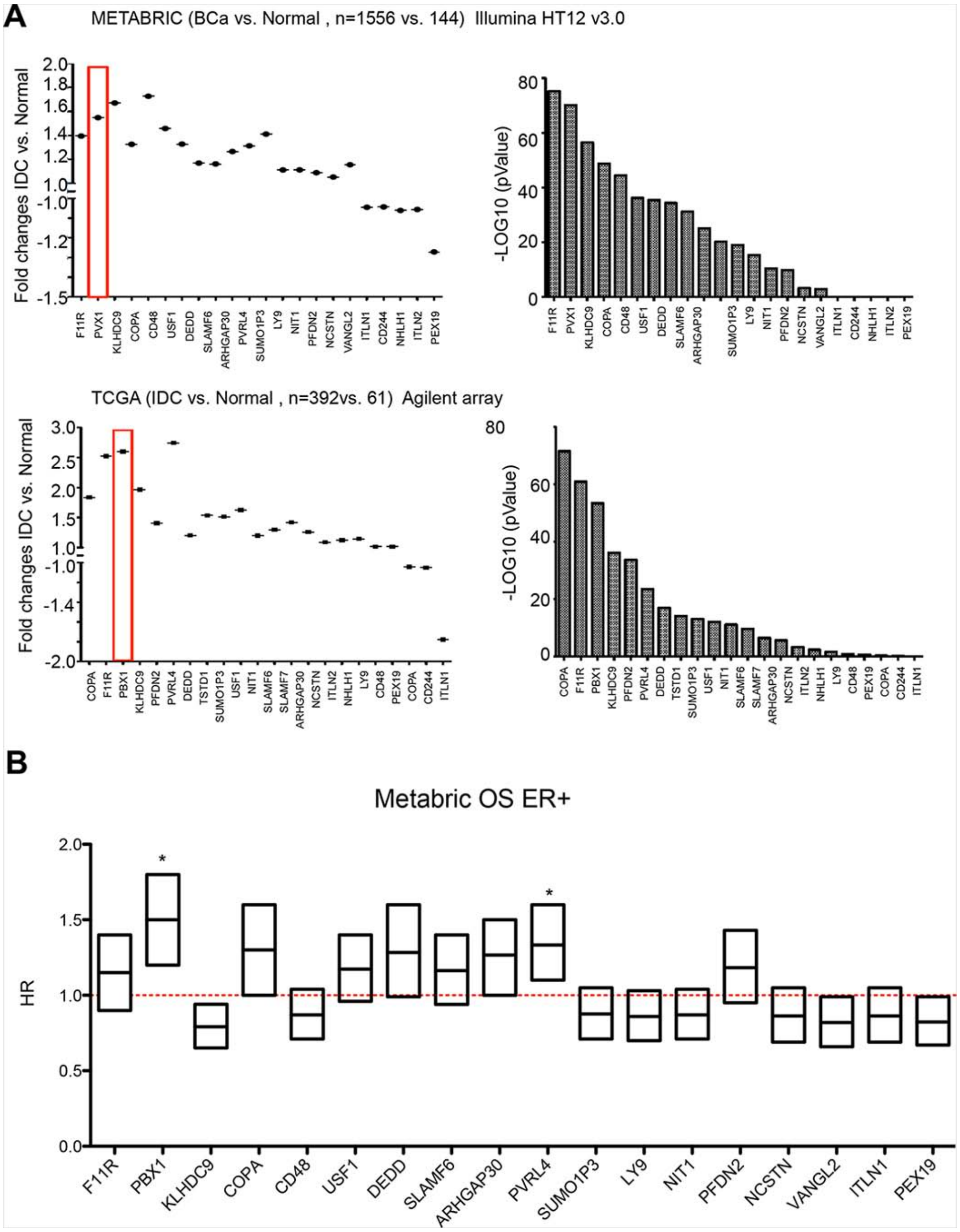

Supplementary Figure S10: A. Transcriptional levels of all genes co-amplified with PBX1 compared to normal breast tissue. Right panel represent the associated –LOG (Pval). B. Forest plot of HR values associated with high-expressors for each indicated gene in ERα-positive patients from the METABRIC dataset.

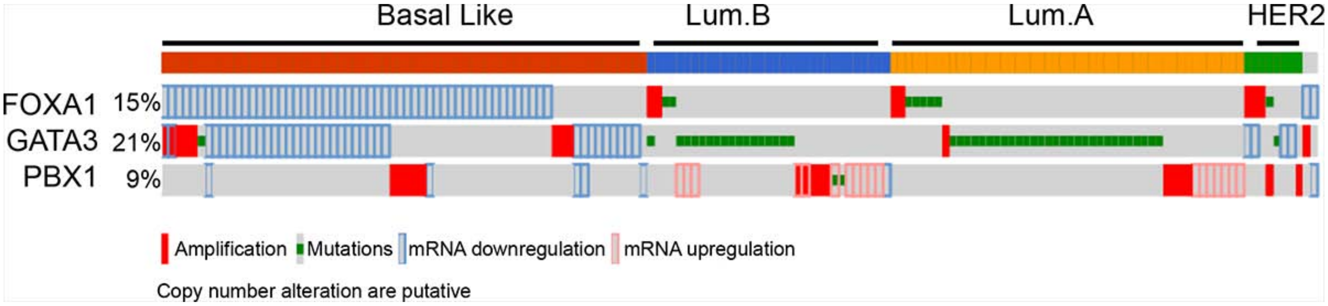

Supplementary Figure S11: Genomics alteration of several ERα pioneer factors using TCGA data [3].

Supplementary Table S1. List of all EGF regulated genes dependent or not from PBX1.

Supplementary Table S2. Detailed Clinico-Pathological and treatment data for patients included in the cfDNA study.
